# Diagnosis and management of rare forms of CAH

**DOI:** 10.1186/1687-9856-2015-S1-O6

**Published:** 2015-04-28

**Authors:** Taninee Sahakitrungruang

**Affiliations:** 1 Chulalongkorn University, Bangkok, Thailand

## 

Congenital adrenal hyperplasia (CAH) is one of the most common inherited metabolic disorders. It comprises a group of autosomal recessive disorders caused by the mutations in the genes encoding for steroidogenic enzymes that are involved in cortisol synthesis [1]. More than 90% of cases are caused by a defect in the enzyme 21-hydroxylase. Four other enzyme deficiencies (P450scc, P450c17, P450c11β, 3βHSD) in the steroid biosynthesis pathway [2-4], along with one cholesterol transport protein defect (StAR) [5], and one electron-transfer protein (P450 oxidoreductase) [6] account for the remaining cases. In these rare forms of CAH, so-called “atypical CAH”, the clinical and hormonal phenotypes can be complicated, and are not well characterized. The clinical symptoms of the different forms of CAH result from the particular hormones that are deficient and those that are produced in excess. A characteristic feature of CAH is genital ambiguity or disordered sex development (DSD), and most variants are associated with glucocorticoid deficiency. This talk will focus on the diagnosis and management of the variant forms of CAH other than 21-hydroxylase so-called “atypical CAH”, including the genetic analyses, and phenotypic correlates.
